# Tricuspid Stenosis Due to Cardiac Sarcoma

**DOI:** 10.7759/cureus.116092

**Published:** 2026-09-11

**Authors:** Mirza M Baig, Muhammad Ansar

**Affiliations:** 1 General Internal Medicine, University Hospitals Plymouth NHS Trust, Plymouth, GBR; 2 Internal Medicine, University Hospitals Plymouth NHS Trust, Plymouth, GBR

**Keywords:** heart failure, pericardial effusion, primary cardiac sarcoma, right atrial mass, tricuspid stenosis

## Abstract

Primary cardiac sarcomas are rare, aggressive malignant tumors with high recurrence rates and poor prognosis. Their initial presentation is often vague, contributing to diagnostic delay and rapid clinical deterioration. We report the case of a 32-year-old man who presented with gastrointestinal symptoms and constitutional features, initially suggesting a hepatobiliary or infectious cause. As his condition progressed, signs of right-sided heart failure emerged, prompting transthoracic echocardiography, which revealed a large right atrial mass causing tricuspid inflow obstruction and a significant pericardial effusion. Surgical resection confirmed biphasic synovial sarcoma, a subtype previously described in small case series. Despite an initially uncomplicated postoperative course, he subsequently developed atrial flutter with associated left ventricular dysfunction, necessitating cardioversion. Anthracycline toxicity remains a possible contributing factor. This case underscores the importance of maintaining a broad differential diagnosis in young patients with unexplained systemic symptoms and highlights the diagnostic value of early echocardiographic assessment when right-sided cardiac involvement is suspected. It also reinforces the need for multidisciplinary management given the tumor’s aggressive behavior and potential for rapid clinical decline.

## Introduction

Primary cardiac sarcomas account for approximately 1% of all soft-tissue sarcomas and represent the most common malignant primary cardiac tumor, with an estimated incidence of 0.001-0.03% [1]. Despite their rarity, they are clinically significant due to their aggressive behavior, rapid progression, and tendency to present late. These tumors can arise in any cardiac chamber and occur across a wide age range, although the mean age at diagnosis is around 40 years. Angiosarcomas and unclassified sarcomas comprise most cases, with angiosarcomas typically involving the right side of the heart.

Synovial sarcoma, although exceptionally rare within the heart, exists in three recognized histological subtypes: monophasic, biphasic, and poorly differentiated, each with distinct morphological patterns and prognostic implications. These subtypes are well described in contemporary literature, including recent analyses in Frontiers in Oncology (2024), and are relevant when considering the spectrum of intracardiac synovial sarcoma presentations [2].

The clinical presentation of cardiac sarcomas is often nonspecific, reflecting the tumor’s location, size, and pattern of growth [2,3]. Symptoms may include dyspnea, chest discomfort, abdominal pain, constitutional features, or signs of heart failure. Importantly, right-sided heart failure symptoms can be subtle and easily misattributed, particularly when accompanied by systemic features such as fever, weight loss, or anorexia. The combination of fever and right-sided cardiac symptoms should prompt clinicians to consider right-heart obstructive pathology, including intracardiac sarcomas, especially in younger patients without conventional cardiovascular risk factors. Diagnosis is frequently delayed until hemodynamic compromise or obstructive phenomena develop. Early use of transthoracic echocardiography is therefore crucial, particularly when unexplained right-sided cardiac features emerge.

Given their aggressive nature, cardiac sarcomas require multidisciplinary management involving cardiology, cardiac surgery, oncology, and advanced imaging [3]. Even with complete surgical resection, recurrence is common, and overall prognosis remains poor [4]. This case highlights the diagnostic challenges posed by these tumors and reinforces the need to consider cardiac malignancy in young patients presenting with unexplained systemic symptoms and evolving signs of right-sided heart failure.

## Case presentation

A 32-year-old South Asian man, previously well, presented three weeks after arriving in the UK with abdominal pain, anorexia, nausea, vomiting, and intermittent fevers. He had no past medical history and no alcohol or tobacco use. Examination revealed jaundice and abdominal distension with ascites. The initial gastroenterology assessment focused on hepatobiliary and infectious causes. Blood tests demonstrated markedly deranged liver function, leukocytosis, and elevated inflammatory markers. Chest radiography showed cardiomegaly, while abdominal ultrasound confirmed ascites without organomegaly (Figure 1).

**Figure 1:**
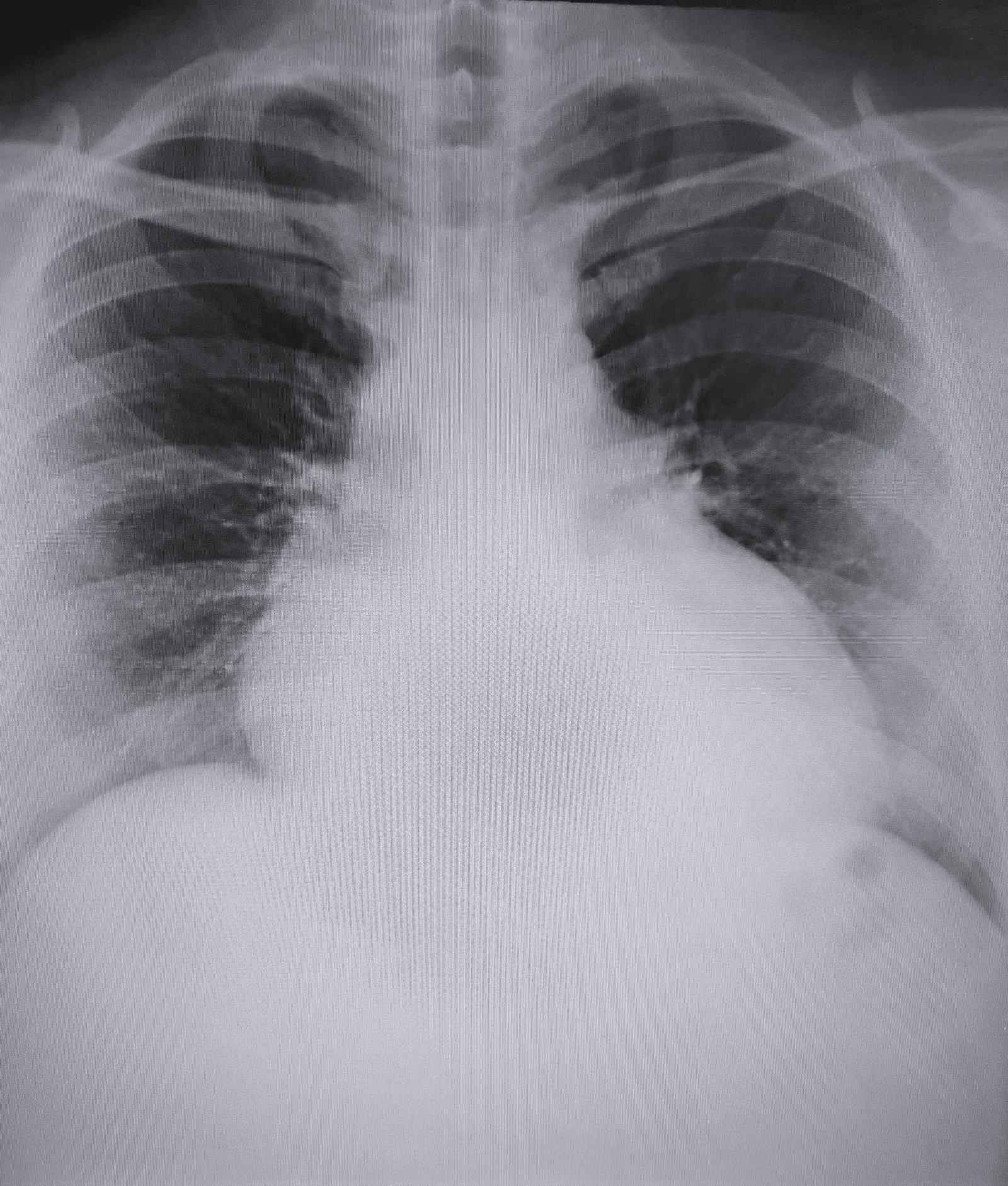
Chest X-ray revealing cardiomegaly secondary to pericardial effusion.

Over subsequent days, he developed a persistently engorged jugular vein and ongoing fever. These evolving cardiac signs prompted transthoracic echocardiography, which identified a large right atrial mass (8 × 3.7 cm) extending through the tricuspid valve into the right ventricle, causing significant diastolic inflow obstruction (Figure 2, apical four-chamber view, arrow pointing at the mass; Figure 3, parasternal long-axis view). A moderate pericardial effusion was also present.

**Figure 2:**
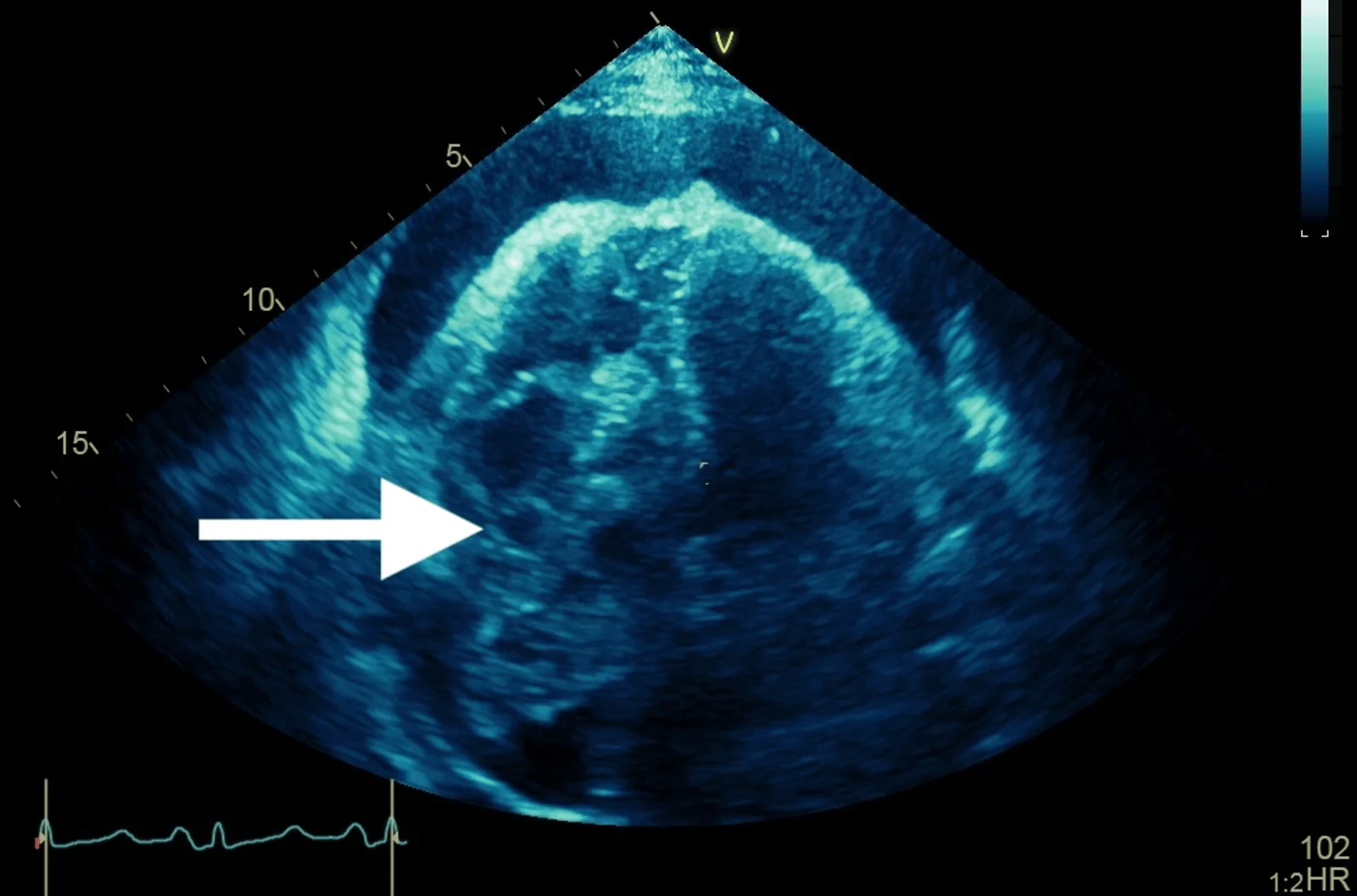
Transthoracic echocardiography apical four-chamber view. The arrow points to the right-sided mass in the right ventricle and right atrium, obstructing the tricuspid valve. Pericardial effusion is noted.

**Figure 3:**
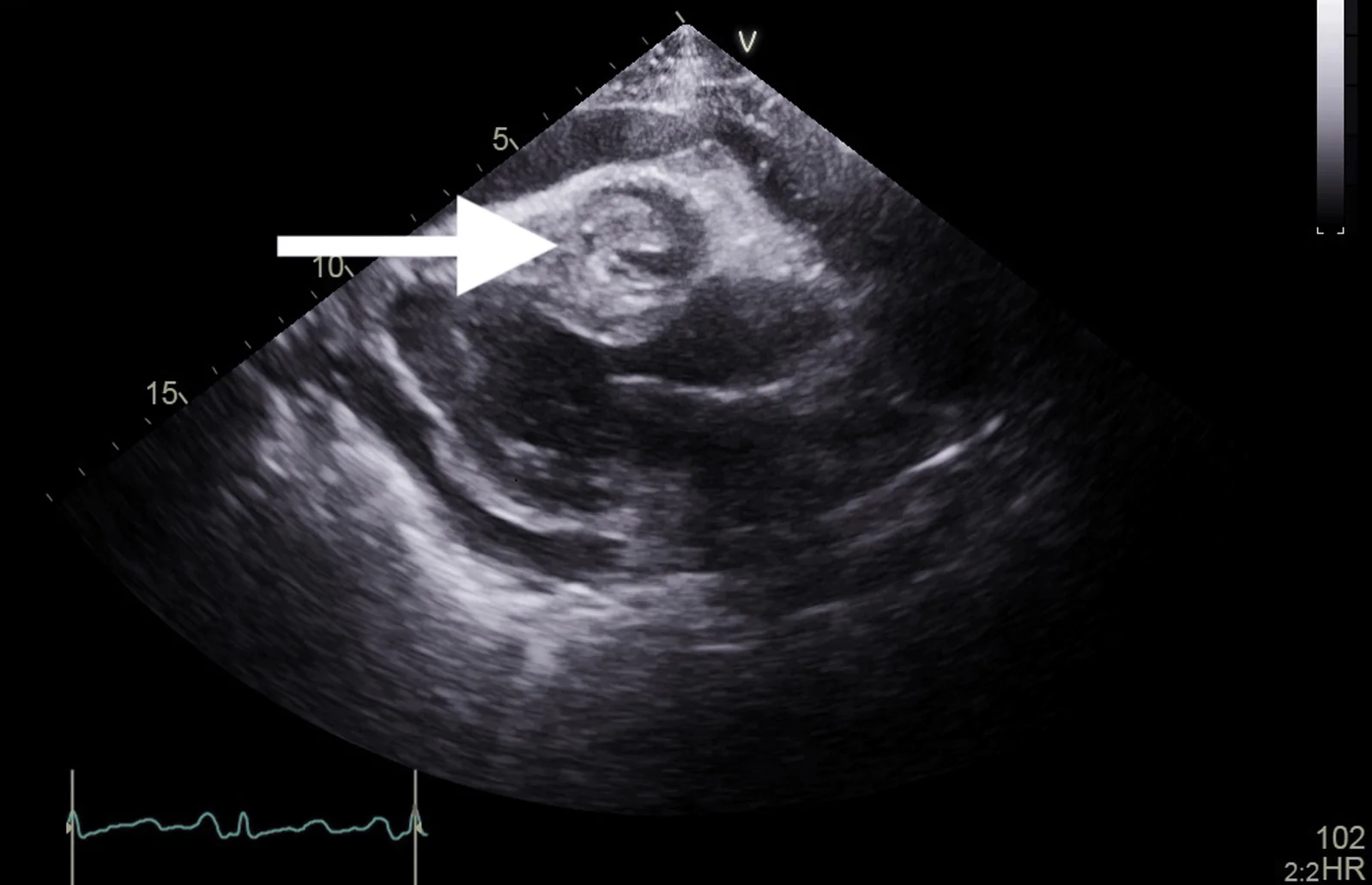
Transthoracic echocardiography parasternal long-axis view. The arrow reveals a mass in the right ventricle.

CT chest imaging confirmed tumor extension into the right ventricle, inferior vena cava, and coronary sinus, and he was transferred to a cardiac surgical center for further management (Figure 4). Unfortunately, neither cardiac MRI nor PET-CT was performed at the surgical center, which limited further characterization of the mass and assessment for metastatic or metabolically active disease.

**Figure 4:**
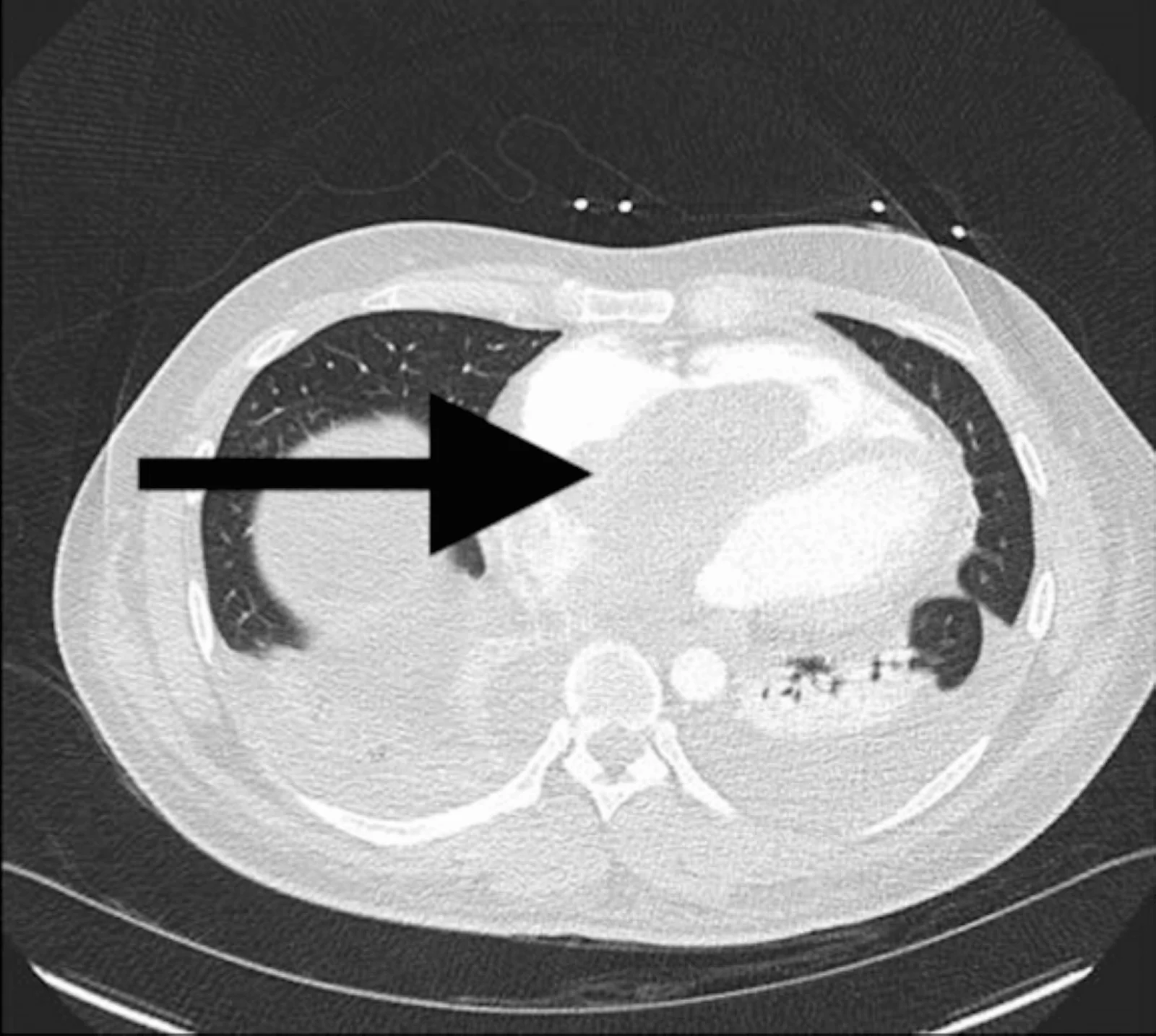
CT chest. Very large intracardiac mass in the right atrium and ventricle.

Differential diagnosis

Early differentials included viral hepatitis, tropical infections, gastrointestinal malignancy, and sepsis, reflecting his initial systemic symptoms of fever, anorexia, weight loss, and abdominal discomfort. As the clinical picture evolved, the combination of systemic symptoms and emerging features of right-sided heart failure, including venous congestion and a persistently engorged jugular vein, became increasingly significant. This constellation should prompt clinicians to consider both cardiac causes of right-sided failure and systemic conditions presenting with constitutional symptoms.

With the development of overt cardiac signs, right-sided heart failure and the possibility of an intracardiac mass became the leading considerations, consistent with intracardiac tumor evaluation. Differential diagnoses at this stage include primary cardiac tumors, metastatic cardiac involvement, infective endocarditis with mass-like vegetations, thrombus, and masses such as lipomas or myxomas.

Treatment

Heart failure was managed with diuretics and careful fluid balance. Pericardiocentesis was considered but was unsuitable due to the posterior location of the effusion. At the surgical center, a pericardial drain was placed, and the case was reviewed in cardio-oncology and sarcoma multidisciplinary meetings.

He underwent extensive right atriotomy. A large mixed tumor was identified, arising near the tendon of Todaro and extending into the inferior vena cava, across the tricuspid valve, and into the right ventricular outflow tract. Additional deposits were found near the superior vena cava and within the left atrium. Complete macroscopic resection was achieved without structural compromise. Histology confirmed biphasic synovial sarcoma.

Outcome and follow-up

He commenced chemotherapy with doxorubicin, ifosfamide, and mesna. After two cycles, he developed new breathlessness and atrial flutter. Echocardiography revealed severe global left ventricular dysfunction (ejection fraction 25-30%). He underwent transesophageal-guided cardioversion with restoration of sinus rhythm on electrocardiography (Figure 5). The cardiomyopathy was suspected to be tachycardia-mediated, although anthracycline toxicity remained possible (cumulative dose 423 mg/m²). He completed four chemotherapy cycles and remains largely symptom-free, with a repeat echocardiogram planned.

**Figure 5:**
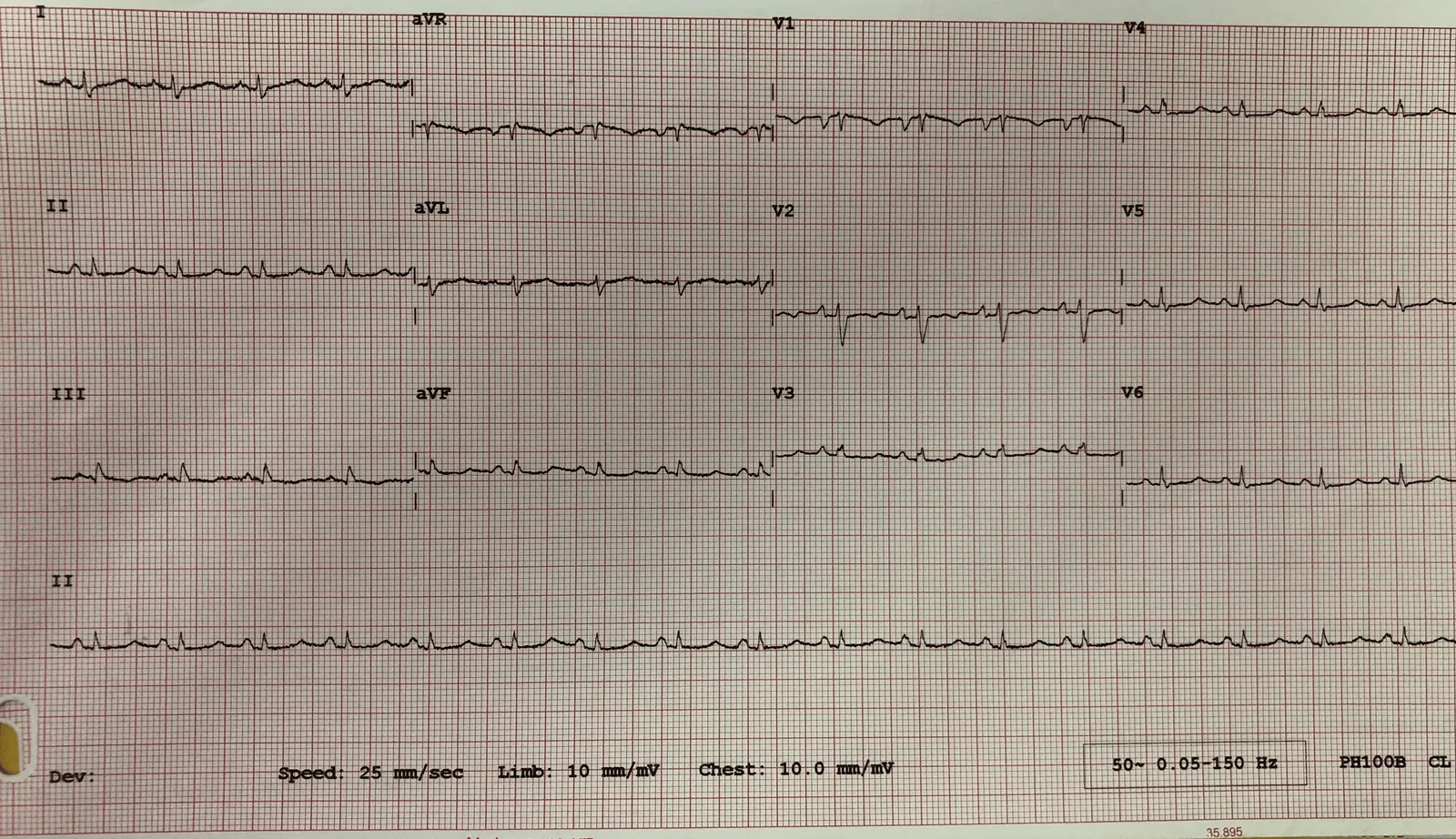
Electrocardiogram revealing sinus tachycardia with low-voltage complexes.

Limitations

A key limitation in this case was the absence of PET-CT imaging, which would have aided metabolic characterization and assessment for extracardiac disease. Similarly, cardiac MRI, the preferred modality for detailed soft-tissue evaluation, was not performed at the surgical center. The lack of these advanced imaging modalities restricted comprehensive tumor staging and tissue characterization.

## Discussion

Primary cardiac sarcomas are exceptionally uncommon malignant tumors, with an estimated autopsy incidence of 0.001-0.03% [1]. Although they constitute the most frequent primary malignant neoplasms of the heart, their clinical presentation is typically vague and often leads to diagnostic delay. In this case, the patient initially exhibited abdominal pain, anorexia, nausea, vomiting, and fever, features that closely resemble the nonspecific systemic manifestations described in the literature, where cardiac sarcomas may present with dyspnea, chest pain, abdominal pain, weight loss, and fever. As the illness progressed, the combination of systemic symptoms and emerging signs of right-sided congestion, including venous engorgement and peripheral edema, became clinically significant. This pattern should raise suspicion for right-heart obstructive pathology, including intracardiac sarcomas, particularly when systemic inflammatory features coexist with signs of impaired right-sided cardiac output. This overlap explains why early evaluation focused on gastrointestinal and infectious causes before cardiac involvement became apparent.

The tumor’s extension from the right atrium through the tricuspid valve into the right ventricle, inferior vena cava, and coronary sinus reflects the infiltrative pattern described by Siontis et al., who noted a high propensity for local invasion and distant metastasis in primary cardiac sarcomas [1]. Angiosarcomas represent the most common subtype of cardiac sarcoma and are usually right-sided, whereas synovial sarcomas are considerably rarer and less well characterized. Synovial sarcoma comprises three histological subtypes: monophasic, biphasic, and poorly differentiated. The monophasic form contains predominantly spindle cells, the biphasic variant demonstrates both spindle and epithelial components, and the poorly differentiated subtype is characterized by high-grade, primitive-appearing cells with aggressive behavior. These subtypes are well described in contemporary literature, including recent analyses in Frontiers in Oncology (2024), and are relevant when considering the spectrum of intracardiac synovial sarcoma presentations [2].

Despite multimodal therapy, prognosis for primary cardiac sarcomas remains poor, with median survival typically less than one year due to recurrence and metastatic spread [1,3]. Talbot et al. describe cases where resection was not possible, necessitating combined heart-lung transplantation [4]. Surgical resection is the preferred initial treatment when feasible, although complete excision is frequently challenging due to extensive infiltration [5]. In contrast, this patient underwent successful extensive resection without compromising native cardiac structures, despite tumor involvement of multiple chambers and venous structures. Postoperative chemotherapy with doxorubicin and ifosfamide aligns with established sarcoma regimens; however, the subsequent development of severe left ventricular dysfunction raises concern for anthracycline-induced cardiomyopathy. Although tachycardia-mediated cardiomyopathy secondary to atrial flutter remains a plausible alternative explanation, the cumulative anthracycline dose of 423 mg/m² places the patient within a recognized risk range for cardiotoxicity.

Echocardiography remains central to the initial identification of intracardiac masses once cardiac symptoms emerge. In this case, transthoracic echocardiography demonstrated a large right atrial mass causing functional tricuspid stenosis and right-sided heart failure. This finding is consistent with the observations of Uzar et al., who emphasize the diagnostic value of echocardiography in detecting cardiac tumors when obstructive physiology is suspected [6,7]. The presence of pericardial effusion, also noted on imaging, is a recognized but variable feature of cardiac sarcomas and may contribute to hemodynamic compromise.

The patient’s current clinical stability and restoration of sinus rhythm are encouraging, yet ongoing surveillance is essential given the high recurrence rates reported across multiple series. Overall, this case illustrates several important themes: the diagnostic challenge posed by nonspecific systemic symptoms, the importance of early echocardiography in young patients with unexplained right-sided heart failure, and the critical role of multidisciplinary cardio-oncology collaboration in managing rare cardiac malignancies.

## Conclusions

This case illustrates the considerable diagnostic difficulty associated with primary cardiac sarcomas, which frequently present with nonspecific systemic symptoms and may initially be mistaken for gastrointestinal or hepatobiliary disease. In this patient, early clinical attention was directed toward abdominal and hepatic pathology, and cardiac involvement only became apparent once signs of progressive right-sided venous congestion emerged. Importantly, the combination of fever and right-sided heart failure features, both of which can be clinically nonspecific, should heighten suspicion for right-heart obstructive tumors, particularly when systemic symptoms coexist with evidence of impaired right-sided cardiac output.

Transthoracic echocardiography played a decisive role in identifying the obstructive right atrial mass and remains an essential first-line, noninvasive modality when intracardiac pathology is suspected. Although macroscopic tumor resection was achieved and multidisciplinary management coordinated effectively, the aggressive nature of synovial sarcoma underscores the importance of early recognition and sustained surveillance. Our longest follow-up to date is nine months. This case emphasizes the need for clinicians to consider intracardiac tumors in young patients presenting with unexplained right-sided heart failure, particularly when accompanied by systemic symptoms such as weight loss, anorexia, and fever, which may serve as early indicators of an underlying cardiac malignancy.
